# Can artificial ecological islands alter the biodiversity of macroinvertebrate? A case study in Fujin National Wetland Park, the Sanjiang Plain, China

**DOI:** 10.1002/ece3.8183

**Published:** 2021-10-05

**Authors:** Zi‐Ao Yuan, Xin‐xin Liu, Hai‐rong Du, Ming‐Hai Zhang

**Affiliations:** ^1^ College of Wildlife and Protected Area Northeast Forestry University Harbin China

**Keywords:** artificial ecological island, biodiversity, China, community composition, ecological restoration, macroinvertebrates, the Sanjiang Plain, wetland park

## Abstract

1. Many policies and studies globally have highlighted the pivotal role of wetland ecosystems regarding wetland biota and their ecological status. With the strengthening of wetland ecosystem management legislation and policy, wetland restoration should also consider increasing habitat diversity to improve biota. We explore whether the construction of artificial ecological islands can increase the diversity of and macroinvertebrates before assessing the effects of actively constructing islands via human intervention on wetland protection.
2. We discuss changes in macroinvertebrate diversity (i) with and without islands, (ii) at different water‐level gradients surrounding the islands, (ⅲ) on different island substrates, and (ⅳ) at different time scales. We used ANOVA, ANOSIM, and cluster analysis to test the differences.
3. The macroinvertebrate communities had spatially heterogeneous distributions which changes over time due to both natural and anthropogenic stresses. The establishment of islands significantly increased the community composition and biodiversity of the macroinvertebrate. Water depth and substrate affect community composition of macrozoobenthos. The abundance and diversity of macroinvertebrates can influence the biodiversity of their predators (fish and waterbirds). Potentially, the construction of islands could provide some cobenefits for the conservation of wetland fauna.

*Synthesis and applications*. Establishing artificial ecological islands in broad open‐water areas and increasing water‐level gradient and substrate diversity can increase microhabitat availability and habitat heterogeneity. These changes can adapt to different ecological niches of aquatic organisms, increase biodiversity, and have a positive effect on the ecological restoration of inland freshwater marshes and wetlands.

## Graphical abstract

This paper proves that it is feasible to build artificial ecological islands in open water to increase the microhabitat heterogeneity and water‐level gradient as a means of ecological restoration, so as to provide more suitable habitats for aquatic organisms and thus increase the biological diversity of the ecosystem. Macrobenthos are the main food for waterfowl and fish. By comparing the effects of islands on macrozoobenthos biodiversity, we found that islands have a positive effect on macrozoobenthos biodiversity by comparing the effects of islands on macrozoobenthos biodiversity. Over time, it will attract more predators, such as fish and waterfowl, and achieve the goal of ecological restoration.

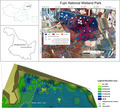

## 1 INTRODUCTION

Wetlands are important components of the natural landscape for their functions in cleaning and retaining water naturally and providing habitats and food sources for a wide variety of plant and animal species. Water consumption continues to increase due to economic development, population growth, and intensive agriculture (croplands). Increased urbanization and infrastructure development, disease control (particularly mosquitoes), and aquaculture continue to convert and degrade wetlands worldwide (Jiang et al., 1998). Historical reports show that 87% of the world's natural wetland area has disappeared since the beginning of the 18th century (Davidson, 2014). Furthermore, between 1970 and 2008, the natural Wetland Extent Trends index range (excluding constructed wetlands) declined by approximately 30% (Dixon et al., 2016). Losses of natural inland wetlands have been consistently greater, and at faster rates, than of natural coastal wetlands. The severe loss of wetlands worldwide has significantly increased the threat to wetland‐dependent organisms (Gregory & Strien, 2010). Especially in China, human activities have severely reduced and modified the original wetland habitats. In the past 25 years, the population density of common amphibians in 29 provinces of China showed a decreasing trend, which was 51.68% (77/149). Amphibians, in particular, which live in freshwater ecosystems, have seen a sharp decline in diversity. In addition, China is an important stopping point for birds along the East Asia–Australia migration route. Wetland destruction has led to a significant decline in wintering waterbird diversity in inland waters and marshes (Nanjing Institute of Environmental Sciences, 2018). To counter these negative trends, many wetland conservation and restoration projects worldwide aim to improve the wetland biome and increase the diversity of wetland‐dependent organisms (Platteeuw et al., 2010). How to increase wetland biodiversity rapidly is a hot topic in global discussions.

In China, the Basic State Policy for the Construction of Ecological Civilization (18th CPC National Congress, 2012) emphasizes the key role of wetland ecosystems and the ecological status of wetland biota. And, in January 2015, the State Council approved the “biodiversity protection major project implementation plan (2015–2020),” emphasized the “implementation of ecological restoration engineering, coastal and inland wetland ecological restoration and strengthening comprehensive management, wetland ecological compensation mechanism, expand the wetland area, especially in the migratory birds move flying route priority in the wetland ecological restoration engineering.” Therefore, protecting the existing wetlands, creating and restoring the functions of degraded wetlands, and their supporting aquatic environment are all important strategic fields in the construction of ecological civilization. For wetland biological management, this importance leads to the goal of attracting a diverse and rich aquatic community from adjacent habitats. It has been reported that the presence of vegetation and associated epiphytic biota in the habitat adds additional nutritional resources to the base of the food web (Lubbers et al., 1990). This vegetation also increases the productivity of fish and invertebrates by increasing food availability and reducing the risk of predation (Clynick et al., 2013; Culler et al., 2014; Irlandi et al., 1995). Following this, depth, flow velocity, substrate, and vegetation affect the abundance and distribution of fish and invertebrates (Al‐Sayed et al., 2008; Hintz et al., 2016). And water depth limits the birds' access to food resources such as fish, invertebrates, and aquatic plants. Therefore, controlling the survival condition factors such as water depth, substrate, and vegetation type of wetlands is a valuable tool. This provides habitat for a variety of species, which can improve the survival and reproductive success of individual organisms (Baschuk et al., 2012; Masero et al., 1999; Mieczan et al., 2014).

Constructing artificial ecological islands (islands for short) is an important technique for improving wetland topography and providing a diversity of foraging depths for wetland fauna (Burton et al., 1996; Wang et al., 2014). In order to increase the biodiversity of Fujin National Wetland Park (park for short), ecological managers have established artificial ecological islands in open‐water area to increase the diversity of microhabitats. However, nearly seven years after the islands were constructed, it is still not known whether the technology has protected or increased biodiversity. Macroinvertebrates are primarily food components of waterfowl and fish with overall abundance and diversity of macroinvertebrates both directly, and indirectly, affecting the biodiversity of predators (Patra et al., 2010). Therefore, we chose macroinvertebrate as experimental subjects to explore the effect of artificial island construction. For comparative purposes, we surveyed differences in macroinvertebrate species richness and abundance between microhabitats including artificial islands. The purpose of this research is twofold: first, determine whether the construction of artificial ecological island will increase the number and diversity of macrobenthos and second, assess whether the substrate, construction age, and water depth of artificial ecological islands have different effects on the increase of macrobenthic diversity. The general hypothesis tested was that the construction of artificial ecological islands increased the number and diversity of related aquatic life. Specifically, it is predicted that the number and growth potential of macrozoobenthos will be greater than that of open‐water areas without ecological islands, and the impact of islands constructed in different substrates and times on the diversity of macrozoobenthos will be different. This paper presents a technical example of wetland restoration project in Sanjiang Plain. This provides technical support and scientific basis for animal protection, biodiversity increase, and wetland protection and utilization in Sanjiang Plain.

## 2 MATERIALS AND METHODS

### 2.1 Study area and artificial ecological islands

China has 65,940 km^2^ of wetlands, spanning multiple latitudes and accounting for about 10% of the world's wetland area, with abundant habitat types, species, and quantities of biological resources. The Sanjiang Plain, located in northeast China, is the largest concentrated distribution area of freshwater marshes in China. It is not only an important ecological resource and environmental protection barrier, but also an important stopover site for many Palaearctic‐realm migratory waterbird species. In past years, the wetland resources in Sanjiang Plain have been seriously degenerated or lost due to long‐term excessive and unreasonable utilization and development. From 2000 to 2015, the total wetland area in Sanjiang Plain decreased by 2508.56 km^2^, and the wetland vegetation coverage rate decreased from 91.8% to 74.0%. This indicates a significant decrease in the supporting capacity of suitable habitats for aquatic organisms (He et al., 2017; Liu et al., 2018).

Fujin National Wetland Park, which covers an area of 22 km^2^, is located in the hinterland of the Sanjiang Plain, Heilongjiang Province, Northeast China (E 131°41′02.8″–131°46′09.2″, N 46°53′18.8″–46°56′18.5″). The wetland area is 12 km^2^, accounting for 54.6% of the total area (National Wetland Park refers to a specific area approved by the state forestry administration and protected and managed in accordance with relevant regulations for the purpose of protecting wetland ecosystem, making rational use of wetland resources, carrying out wetland publicity, education, and scientific research) (National Forestry & Grassland Administration, 2017). This area has a temperate continental monsoon climate with distinct seasons. There is less rain in spring and more in summer, and the temperature drops sharply and differs in autumn. The annual precipitation is approximately 608.6 mm, and the average temperature is −20.4°C in January and 22.2°C in July.

Before 2004, the park's cofferdams were crisscrossed and cultivated, the wetlands were almost all reclaimed, and the wetland resources were severely damaged. In 2005, the local government decided to strengthen the wetland restoration project to comprehensively protect the wetland ecosystem. The ecological restoration of wetlands was carried out by means of water diversion, increasing vegetation diversity and establishing artificial ecological islands. This has become a successful example of the conversion of farmland to forests and wetlands in China. Due to the flat topography of the park and the uniform distribution of various environmental factors, the plant diversity is low, mainly *Phragmites australis* and *Typha*. A single resting and foraging habitat results in very few animal species in the park. To create good habitats for aquatic organisms, attract birds, and increase the integrity of the wetland ecosystem of the park, the local government hired the UK's Wildfowl and Wetland Trust (WWT) and domestic and foreign experts to scientifically plan and design the park. The city also cooperated with the German government for technical and financial purposes regarding wetland biodiversity conservation and ecological environment restoration projects.

**FIGURE 1:**
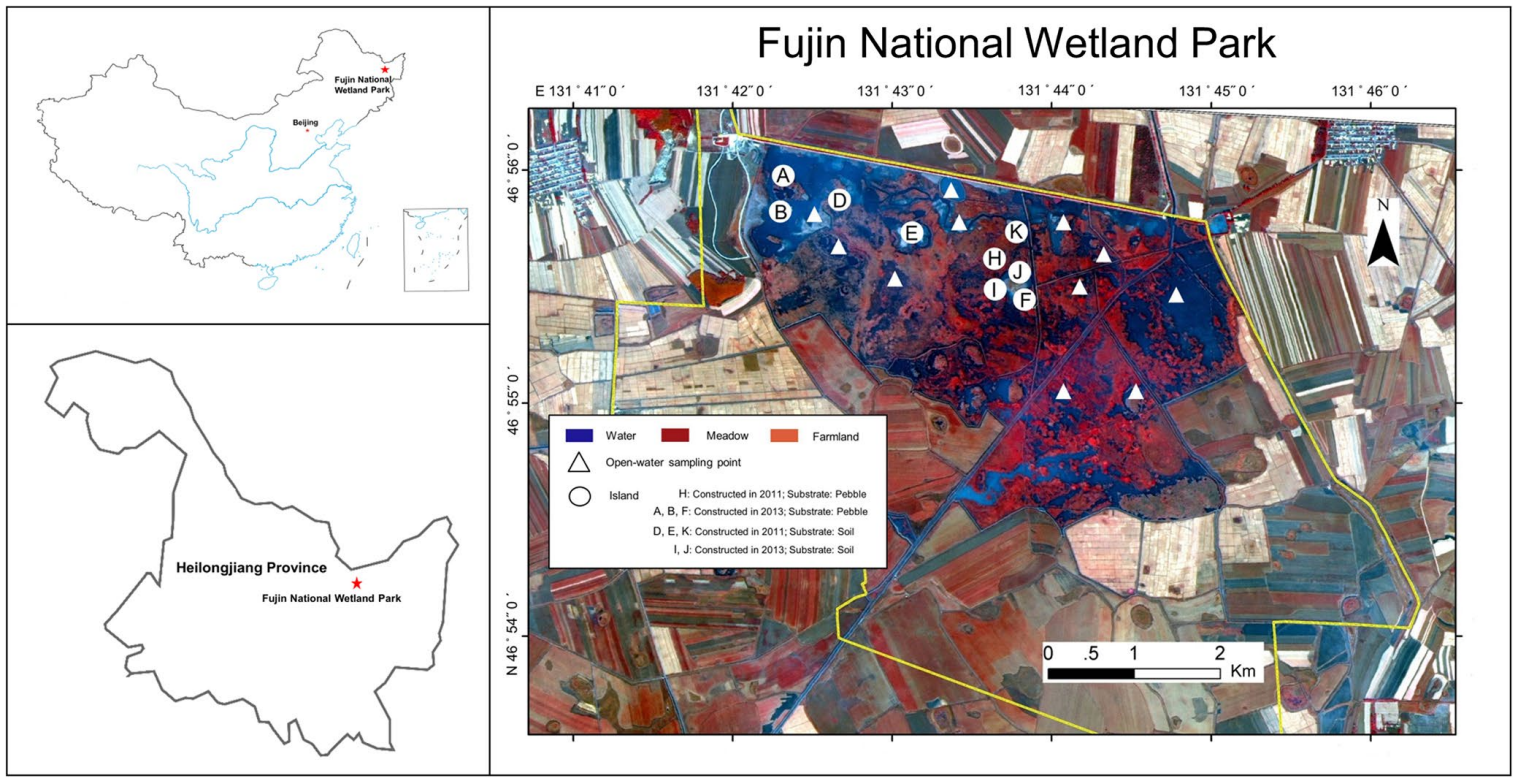
Map of the macrozoobenthos and bird research regions in Fujin National Wetland Park, and basic information of artificial ecological islands

**FIGURE 2:**
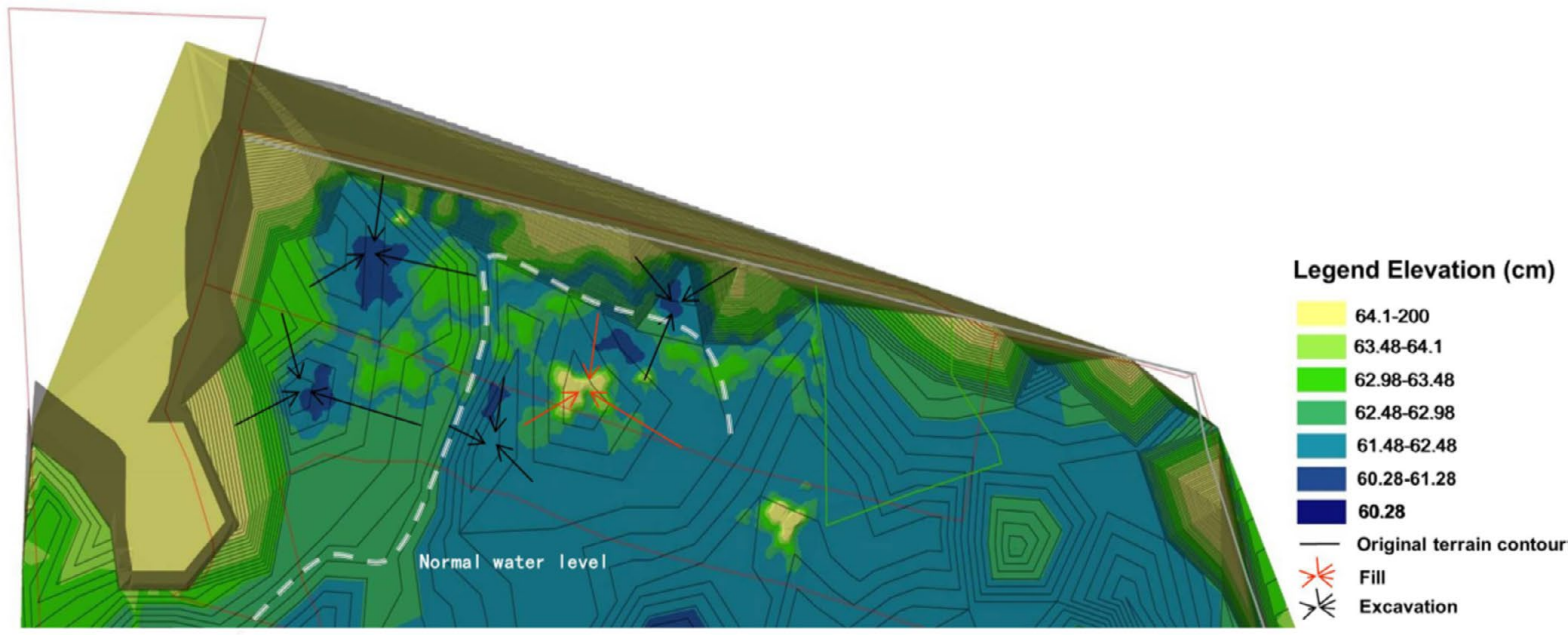
Construction plan of artificial ecological island

**FIGURE 3:**
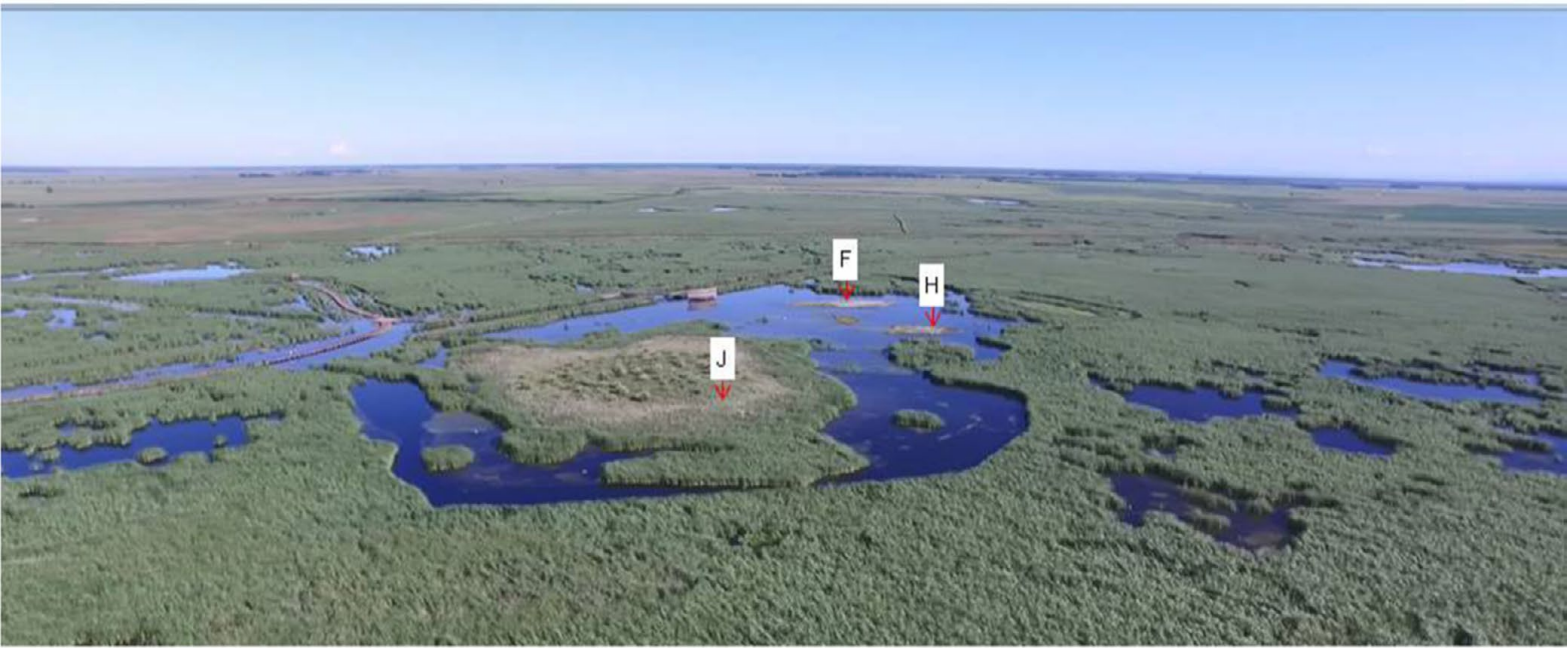
Aerial photo of some of the artificial islands in the park (J, F and H)

In this project, through the construction of ecological island, the purpose of increasing the microhabitat types and enhancing the heterogeneity of various abiotic environmental factors such as hydrology and topography was achieved. These islands can provide habitats for more species of hydrophytes and increase the diversity of plants to improve primary production in wetlands. Wildlife diversity, such as benthic animals and fish, depends on plant growth; plant growth attracts birds that feed on them, and their settling achieves the purpose of having more biological species in a relatively smaller area. Six islands were constructed in the park's open waters in 2011 and 2013, creating a total of 12 islands. To construct the islands, canals were dug in the park; the canals were expanded, and the slope habitat was increased to strengthen the hydraulic connection among water patches. The original low‐lying areas were dug to a depth of more than 2 m according to the terrain, and as a result, the water levels were distributed in different layers and regions that were adapted to the requirements of different overwintering wildlife. Simultaneously, the excavated earth was designed according to the terrain and stacked on relatively higher ground to form soil substrate islands (SIs) above the water. Pebbles were placed on some of the soil islands to form pebble substrate islands (PIs) (Figure 1). As a result, the original plateau is now more prominent, and there is always a certain area of land at the highest water level to achieve significant topographic differences. The island shape is the frustum of a cone, with the highest point of the island rising approximately 1 m above the water surface on average (Figure 2). The edge slope of each island is different, and the shallow water zone is very limited. The total island area was approximately 3 km^2^ after it was built. Due to island collapse, the size of each island that extends out of the water currently varies from 200 to 5000 m^2^ (An aerial photograph of some of the islands is shown in Figure 3).

Topographic changes affect the formation of landscape pattern of the park, resulting in changes in water, soil, and other conditions. This greatly enriches the diversity and distribution range of animals and plants in the park. Ecological islands provide places for animals to find food, breed, and rest. These structures attract waterfowl because they have wide views and are inaccessible to mammalian predators (Momose et al., 1998). Therefore, the structure and function of the wetland ecosystem has been gradually restored, and the biodiversity of the wetland has improved.

### 2.2 Methods

Macroinvertebrate samples were collected in the summer of 2015–2017 (mid‐August). In order to investigate the heterogeneity of island vegetation structure and composition, a field survey was conducted in July–August 2015.

Macroinvertebrate samples were collected in a 1‐m^2^ quadrat from 20 sites with D‐frame kick nets (30 cm aperture, 500‐mm mesh) and then sieved with water. The retained macroinvertebrates were transferred into prelabeled polyethylene containers. The faunal samples were fixed using buffered formalin (4%) and subsequently preserved using 70% ethanol. The organisms were identified and counted to the “species” level (Al‐Sayed et al., 2008). Due to the collapse of some islands, nine islands (Figure 1) were selected. Survey sample points were set on those islands, and two depth gradients were established on each island, namely, a shallow water‐level area (depth < 40 cm) and a deepwater‐level area (40 cm < depth < 80 cm). Samples were randomly collected at each depth gradient, and repeated sampling was conducted in three directions in the shallow water habitat "around" the islands. To prevent disturbance caused by the migration of macroinvertebrates from the islands to the open water, 11 samples were randomly selected from an open‐water area (50 cm < depth < 130 cm) that was far from the islands in the park. The open‐water area is separated from the island sampling sites by deep river channels and dams, thus ensuring the independence of the sampling sites. The distance between the open‐water sampling points depends on the size of the floating raft between the sampling points, ranging from 100 to 300 m. In total, 20 samples were taken (Figure 1).

At each sampling site, we calculated the mean macroinvertebrate abundance and recorded the species. For the benthic communities in each group identified by the cluster analysis, the average density and number of species (considering each taxon as a species) were determined. An initial multivariate analysis was performed using the standardized species matrix in a cluster analysis (Bray–Curtis hierarchical clustering), and nonmetric multidimensional scaling (MDS) was performed using the similarity scores generated from the cluster analysis (Clarke & Warwick, 1994). These analyses were performed to find any “natural groupings” based on the species matrix to check whether the grouping was consistent with the artificial grouping results based on the species matrix (Butcher et al., 2003). We used Q–Q plots of the residuals in SPSS to compare the fit of the common distributions (normal, Poisson, negative binomial). The procedure indicated that the normal distribution fit the data well.

It has been shown that the use of a higher taxon (especially families), as surrogates for species diversity, has been shown to be relevant in freshwater community analyses (Heino & Soininen, 2007; Hewlett, 2000; Viol et al., 2009), focusing on Ephemeroptera, Plecoptera, and Trichoptera, considered that genus‐level and species‐level identification is unnecessary in broad‐scale monitoring, as identification at the family level only is sufficient. Therefore, we conduct the following studies on macrobenthos at the level of family. (It is important to note that the main risk with higher taxa analyses is to find no significant differences between sites while such differences actually exist.) The purpose of this study was to explain the differences in the response of macrobenthos to environmental factors (island or no island, island substrate, water depth, etc.) among different taxa. The comparative analysis among the samples was conducted at the level of “family.” One‐way ANOVA was carried out to compare differences in the macroinvertebrate species abundance among the various sites in 3 years. The taxa abundances were log (*x* + 1)‐transformed to dampen the effects of the few most abundant taxa. In order to compare biodiversity differences between sampling sites more clearly, we calculated the traditional measures of biodiversity at the "species" level, the Shannon–Wiener index (H’, log e) (Shannon., 1948), Margalef index (d) (Margalef., 1958), and Pielou evenness index (J) (Pielou., 1975). Analysis of similarities (ANOSIM) was used to evaluate the community similarity. Moreover, similarity percentage analysis (SIMPER) was used to determine the contributions of individual taxa toward the dissimilarity between and similarity within the groups identified by cluster analysis, both of which were included in the PRIMER V5.2 software package (Clarke & Gorley, 2006; Clarke & Warwick, 1994). Pearson correlation tests were also performed in the IBM SPSS Statistics 20 software package to determine correlations between vegetation, benthic fauna, birds, and visitor numbers.

## 3 RESULTS

### 3.1 Aquatic invertebrate communities of the park

Across 20 sampled sites, we observed a total of 106 species of 34 macroinvertebrate families. We determined 14143 individuals to family level; they belonged to Mollusca (six families, 15 species, 9807 individuals), Arthropoda (26 families, 84 species, 4274 individuals), and Annelida (2 families, 7 species, 62 individuals).

**FIGURE 4:**
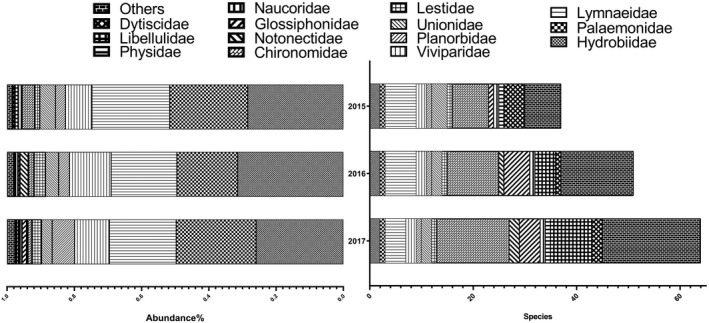
Changes in the number and abundance of major taxa of macrobenthos in the park from 2015 to 2017 (the number of individuals was more than 0.3% of the total)

SIMPER analysis showed that only 3 species were the primary contributors at all sites, *Parafossarulus striatulus* (35.72%), *Palaemon modestus* (34.84%), and *Radi plicatula* (13.49%), with a cumulative contribution of approximately 84% and a total similarity of 38.17 (the list was truncated when 80% was reached). The species and abundance of macroinvertebrate fauna in the park increased continuously in 3 years. The abundance of Chironomidae larvae decreased, but those of Libellulidae and Hydrobiidae increased continuously (Figure 4).

### 3.2 Influence of island type on macroinvertebrate family diversity

**FIGURE 5:**
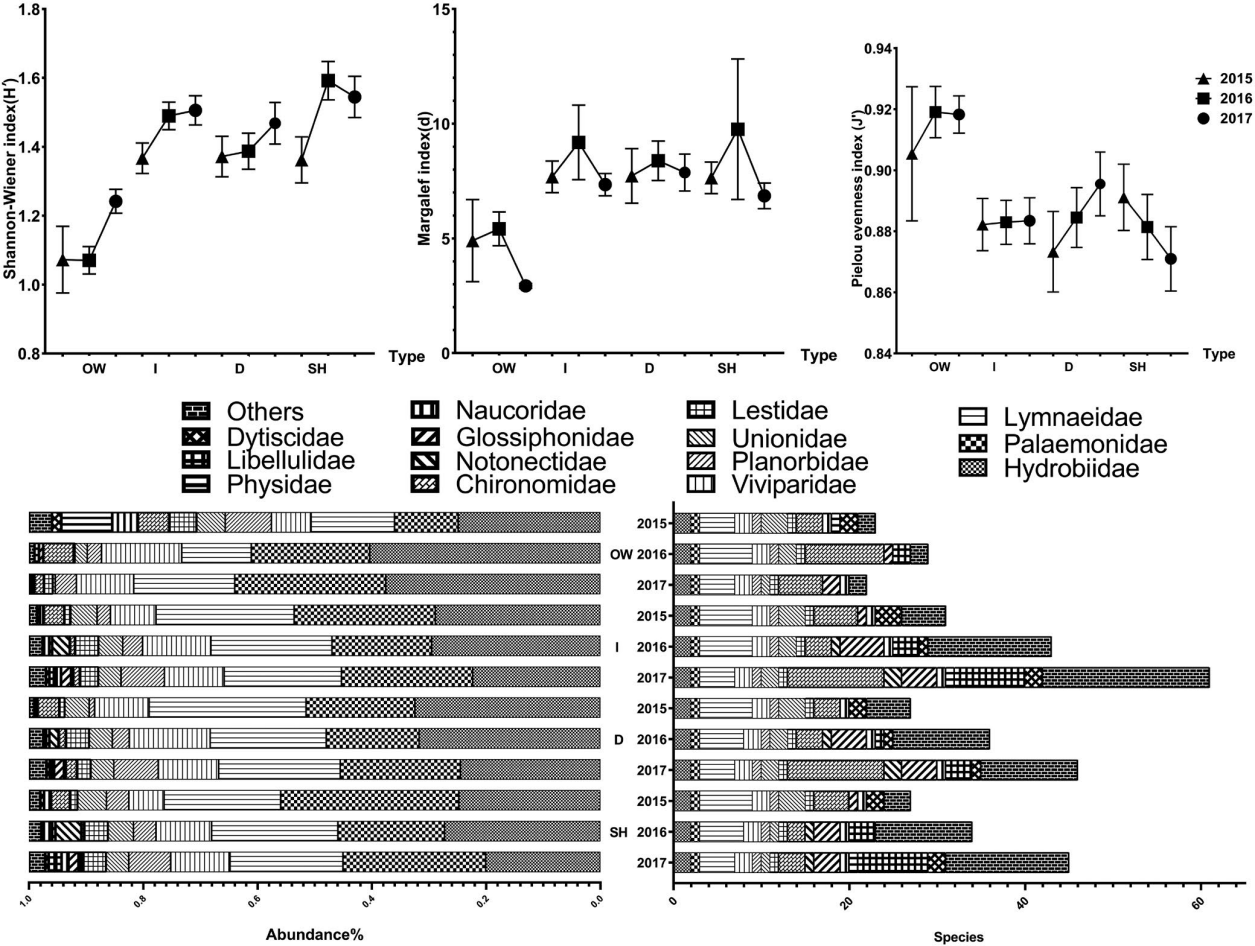
Comparison of the major taxa of macroinvertebrate composition and biotic indices between the islands and the open‐water (the number of individuals was more than 0.3% of the total) (OW: open‐water area; I: islands; SH: shallow water‐level area of island; D: deepwater‐level area of island)

The species abundance and diversity were higher in the island communities than in the open‐water area. Regarding the two water levels on the islands, the richness in the shallow water‐level area was higher but not as evenly distributed as that in the deepwater‐level area (Figure 5). Two‐way crossed ANOSIM showed that there were significant differences among the open‐water area and island two levels regarding the macrobenthos community composition in 3 years (*p* = .001) (Table 1). The water depth of the island was significantly different from that of the open water (*p* = .000). We found that constructing island had an impact on the number of macroinvertebrate in nine families (e.g., Unionidae (*p* = .000), Nepidae (*p* = .000), Lestidae (*p* = .000), and Glossiphoniidae (*p* = .020) had remarkable effects). 28 of these families are significantly abundant in the islands group (e.g., Unionidae, Lymnaeidae, Lestidae, Arachnida, and Glossiphonidae); however, seven families of benthos (e.g., Physidae, Hydrobiidae, and Chironomidae) are significantly abundant in the open water (Table 2).

**TABLE 1:** Two‐way crossed ANOSIM for testing the effects of time (from 2015 to 2017) and sampling sites (open‐water area and islands) on the composition of macrobenthic community

	Global R	Pairwise test R	*p*‐value
Year	0.135		.001*
2015 and 2016		0.086	.001*
2015 and 2017		0.195	.001*
2016 and 2017		0.15	.001*
Site	0.036		.002*
I and OW		0.136	.001*
OW and SH		0.18	.001*
OW and D		0.159	.001*
D and SH		0.022	.001*
I and SH		0.008	.274
I and D		−0.016	.873

Note

(OW: open‐water area; I: islands; SH: shallow water‐level area of island; D: deepwater‐level area of island). The macrobenthic community was significantly changed by time (Global R = 0.135, *p* < .01) and sampling site (Global R = 0.036, *p* < .01).

**TABLE 2:** List of macrobenthic taxa identified in the respective ecological groups in the park

		3 years	Islands & Open water		Water‐level gradients		Deep & Open water		Substrates		Time scales	
		*p*‐Value	*p*‐Value	Abundance trend	*p*‐Value	Abundance trend	*p*‐Value	Abundance trend	*p*‐Value	Abundance trend	*p*‐Value	Abundance trend
Mollusca	Eulamellibranchia											
	Unionidae	.999	.000	I>OW	.988	D<S	.000	D>OW	.917	PI<SI	.001	I < II
	Basommatophora											
	Planorbidae	.000	.054	I>OW	.498	D<S	.127	D>OW	.076	PI>SI	.783	I < II
	Physidae	.033	.180	I<OW	.003	D<S	.085	D<OW	.630	PI<SI	.004	I < II
	Lymnaeidae	.000	.000	I>OW	.051	D>S	.000	D>OW	.038	PI>SI	.668	I > II
	Mesogastropoda											
	Hydrobiidae	.000	.000	I<OW	.000	D>S	.048	D<OW	.000	PI>SI	.001	I < II
Anehropoda	Viviparidae	.000	.898	I>OW	.008	D>S	.267	D>OW	.000	PI>SI	.010	I < II
	Palaemonidae	.000	.310	I>OW	.007	D<S	.896	D<OW	.020	PI>SI	.673	I > II
	Hemiptera											
	Naucoridae	.947	.142	I>OW	.001	D<S	.809	D<OW	.328	PI>SI	.007	I < II
	Gerridae	.424	.606	I>OW	.318	D<S	‐		.318	PI>SI	.410	I < II
	Nepidae	.001	.000	I>OW	.125	D>S	.002	D>OW	.563	PI<SI	.860	I > II
	Notonectidae	.000	.000	I>OW	.008	D<S	.000	D>OW	.683	PI<SI	.808	I > II
	Ephemerptera											
	Siphlonuridae	.102	.371	I>OW	.554	D>S	.158	D>OW	.083	PI>SI	.083	I < II
	Baetidae	.013	.846	I<OW	.045	D>S	.486	D>OW	.083	PI>SI	.083	I < II
	Trichoptera											
	Phryganeidae	.179	.465	I>OW	.991	D>S	.466	D>OW	.800	PI>SI	.319	I > II
	Coleoptera											
	Gyrinidae	.197	.319	I<OW	—		.319	D<OW	—		—	
	Dytiscidae	.012	.146	I>OW	.902	D>S	.186	D>OW	.424	PI>SI	.527	I < II
	Belostomatidae	.579	.002	I>OW	.180	D<S	.083	D>OW	.025	PI>SI	.021	I < II
	Hydrophilidae	.002	.005	I>OW	.982	D>S	.045	D>OW	.062	PI<SI	.020	I > II
	Chrysomelidae	.537	.045	I>OW	.311	D>S	.083	D>OW	.720	PI>SI	.700	I > II
	Scirtidae	.290	.536	I>OW	.231	D>S	.381	D>OW	.231	PI>SI	.231	I < II
	Haliplidae	.001	.000	I>OW	.168	D<S	.045	D>OW	.001	PI>SI	.677	I > II
	Odonata											
	Epiphlebiidae	.197	.606	I>OW	.318	D>S	.466	D>OW	.403	PI<SI	.319	I > II
	cordulegasteridae	.032	.474	I>OW	.185	D<S	.940	D>OW	.278	PI>SI	.046	I < II
	Agrionidae	.534	.470	I<OW	.083	D>S	.580	D<OW	.782	PI<SI	.158	I > II
	Libellulidae	.003	.215	I>OW	.012	D<S	.793	D<OW	.358	PI<SI	.450	I > II
	Lestidae	.001	.000	I>OW	.298	D<S	.002	D>OW	.218	PI>SI	.059	I < II
	Aeschnida	.143	.001	I>OW	.254	D<S	.045	D>OW	.972	PI>SI	.738	I > II
	Diptera											
	Tipulidae	.424	.606	I>OW	.318	D<S	‐		.318	PI>SI	.410	I < II
	Tabanidae	.833	.318	I<OW	.752	D<S	.273	D<OW	.448	PI>SI	.153	I < II
	Stratiomyiidae	.424	.606	I>OW	.318	D<S	—		.318	PI>SI	.410	I < II
	Ephydridae	.075	.371	I>OW	.573	D<S	.466	D>OW	.369	PI>SI	.158	I < II
	Chironomidae	.015	.088	I<OW	.012	D>S	.400	D<OW	.005	PI>SI	.113	I < II
Annelida	Tubificida											
	Tubificidae	.394	.051	I>OW	.156	D>S	.051	D>OW	.546	PI>SI	.903	I > II
	Rhynchobdellida											
	Glossiphonidae	.000	.020	I>OW	.912	D>S	.047	D>OW	.003	PI>SI	.577	I < II

Note

Results of pairwise one‐way ANOVA for the different site types in the park and the abundance comparison to the dissimilarity between groups for each taxa. (OW: open‐water area; I: islands; SH: shallow water‐level area of island; D: deepwater‐level area of island; I: island constructed in 2011; II: island constructed in 2013; PI: pebble substrate island; SI: soil substrate island; and 3 years: Variation in macrobenthic groups for each taxa over a three‐year period.). Two groups of islands constructed at different times (2011 and 2013) also show significant changes in the island's macrobenthic biodiversity over time under the same conditions (weather, water environment).

We also found significant differences in deep and shallow water levels on the islands (*p* = .000), which significantly affected the populations of macrobenthos in eight families (e.g., Viviparidae (*p* = .008), Hydrobiidae (*p* = .000), Palaemonidae (*p* = .007), and Chironomidae (*p* = .012)). Fifteen of these families are significantly abundant in the deepwater levels (e.g., Lymnaeidae, Hydrobiidae, Viviparidae, Nepidae, Chironomidae), while twenty families of benthos (e.g., Palaemonidae, Naucoridae, Notonectidae, Libellulidae, and Lestidae) are significantly abundant in the shallow water levels of islands (Table 2).

In order to investigate the effect of water level heterogeneity on macrobenthos, the populations of macrobenthos in open waters and deep waters of islands were compared. The results showed that water‐level heterogeneity significantly affected the population numbers of 10 families of macrobenthos, such as Eucericidae (*p* = .000), Trionychidae (*p* = .000), Lichthyidae (*p* = .002), and Lichthyidae (*p* = .047) (Table 2). Nine of these families are significantly abundant in the open water (e.g., Physidae, Hydrobiidae, Palaemonidae, Chironomidae), while 26 families of macrobenthos (e.g., Planorbidae, Viviparidae, Notonectidae, and Glossiphonidae) are significantly abundant in the deepwater levels of islands.

**FIGURE 6:**
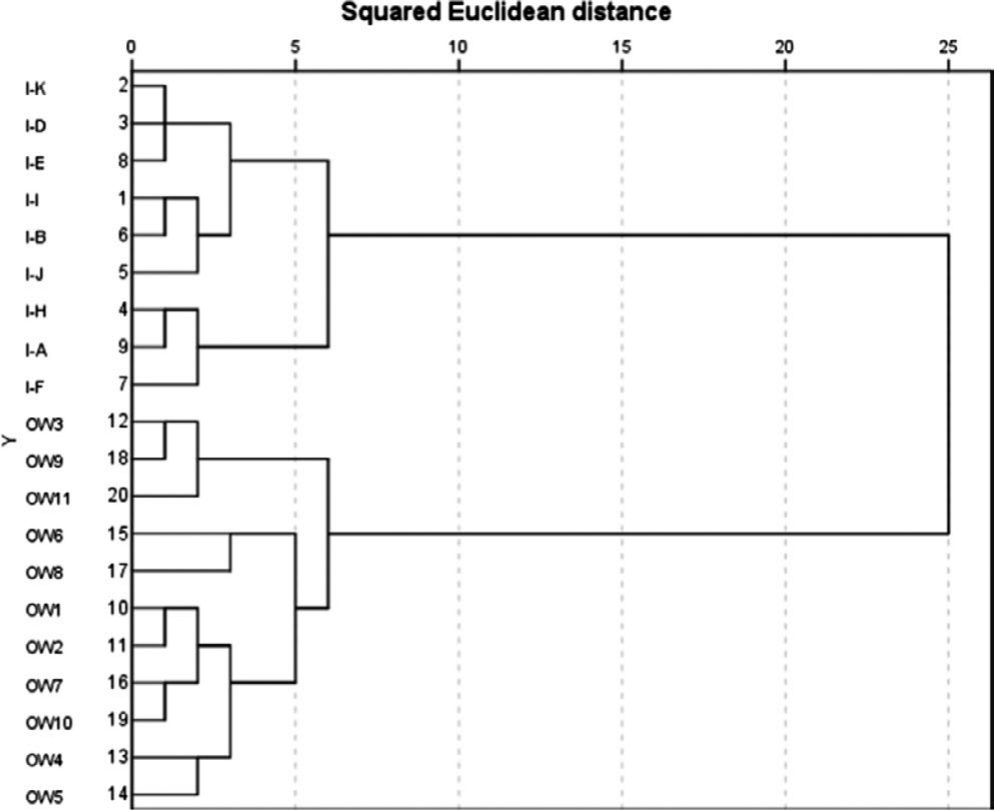
Hierarchical cluster dendrogram of the major taxa of macroinvertebrate biological dataset collected in the park based on the spatial distribution patterns. The sites were grouped into two groups: island and open‐water (OW: open‐water area; I: islands)

**FIGURE 7:**
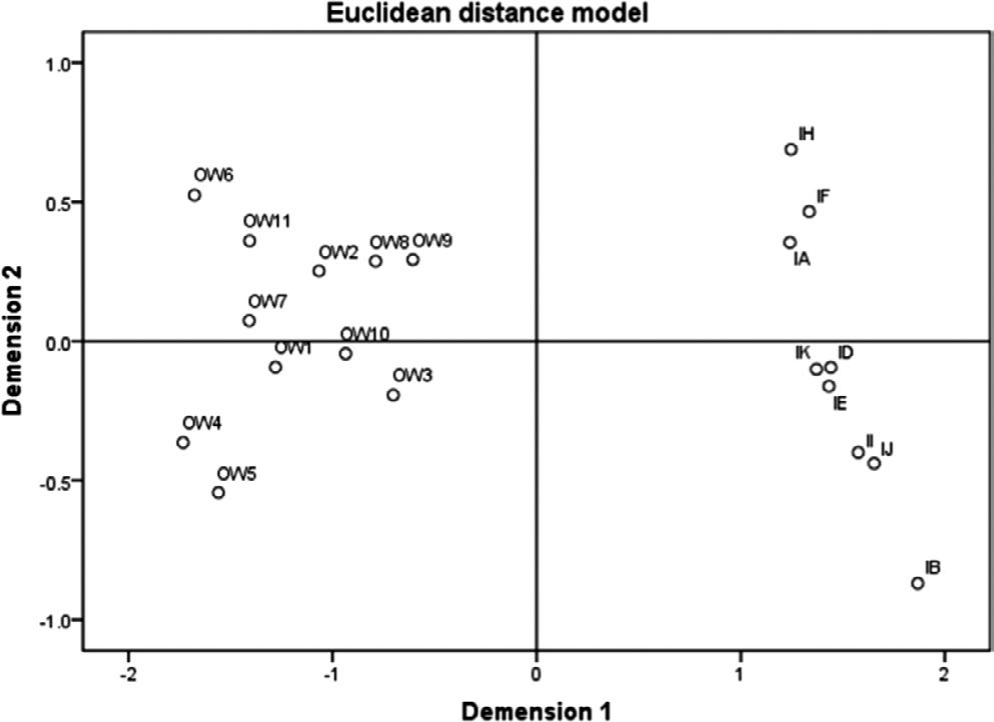
MDS of the twenty macroinvertebrate communities, which were grouped into two groups: island and open‐water area (Stress = 0.051, RSQ = 0.984). In the island group, there were two sub‐groups: PIs (islands H, F and A) and SIs (islands K, D, E, I, J and B) (OW: open‐water area; I: islands)

The hierarchical cluster dendrogram of the 20 macrobenthos communities assessed with SPSS software was basically consistent with the MDS. There were two groups: islands and open‐water area (Figures 6 and 7). The distribution of macroinvertebrate is not uniform due to the wide variation in substrate types, hydrophytes, and depths in open‐water area.

In the MDS results, the island group was divided into two groups (dimension 2), and the division was related to the different substrates. Following two‐way crossed ANOSIM, the community composition differed significantly between the two substrates (*p* = .02) (Table 3). There were significant differences in 8 families between the two groups (e.g., Lymnaeidae (*p* = .038), Palaemonidae (*p* = .020), Belostomatidae (*p* = .025), and Glossiphoniidae (*p* = .003)). (Table 2). The PI groups had more species and were more evenly distributed than the SI. In the classification, PI B was classified as a SI. The possible reason is that island B has a large area, and a few stones were laid around the island. The outlying part of the island is covered with a few pebbles; only the part near the center of the island is covered with more pebbles. It is also covered with *Phragmites australis*, *Typha*, and other vegetation, making it similar to the SI.

**TABLE 3:** Two‐way crossed ANOSIM for testing the islands’ substrate and construction time with year on the macrobenthos communities in the park and the differences in macrobenthos communities between substrate and construction time. Two‐way crossed ANOSIM for testing the effects of time (2015–2017) and island construction time on the composition of macrobenthic community (the left half of the table), as well as the effects of time (2015–2017) and islands' substrate on the macrobenthic community composition (the right half of the table)

	Global R	Pairwise test R	*p*‐value		Global R	Pairwise test R	*p*‐value
Year	0.148		.001*	Year	0.154		.001*
2015 and 2016		0.111	.001*	2015 and 2016		0.103	.001*
2015 and 2017		0.291	.001*	2015 and 2017		0.197	.001*
2016 and 2017		0.138	.001*	2016 and 2017		0.174	.001*
Construction time	0.062		.001*	Substrate	0.042		.02*

Note

The macrobenthic community was significantly changed by time (Global R = 0.148, *p* < .01, year (construction time); Global R = 0.154, *p* < .01, year (Substrate)). The community composition of macrobenthic fauna of islands with different construction times (Global R = 0.062, *p* < .01) and different substrates (Global R = 0.042, *p* < .05) was significantly different.

**FIGURE 8:**
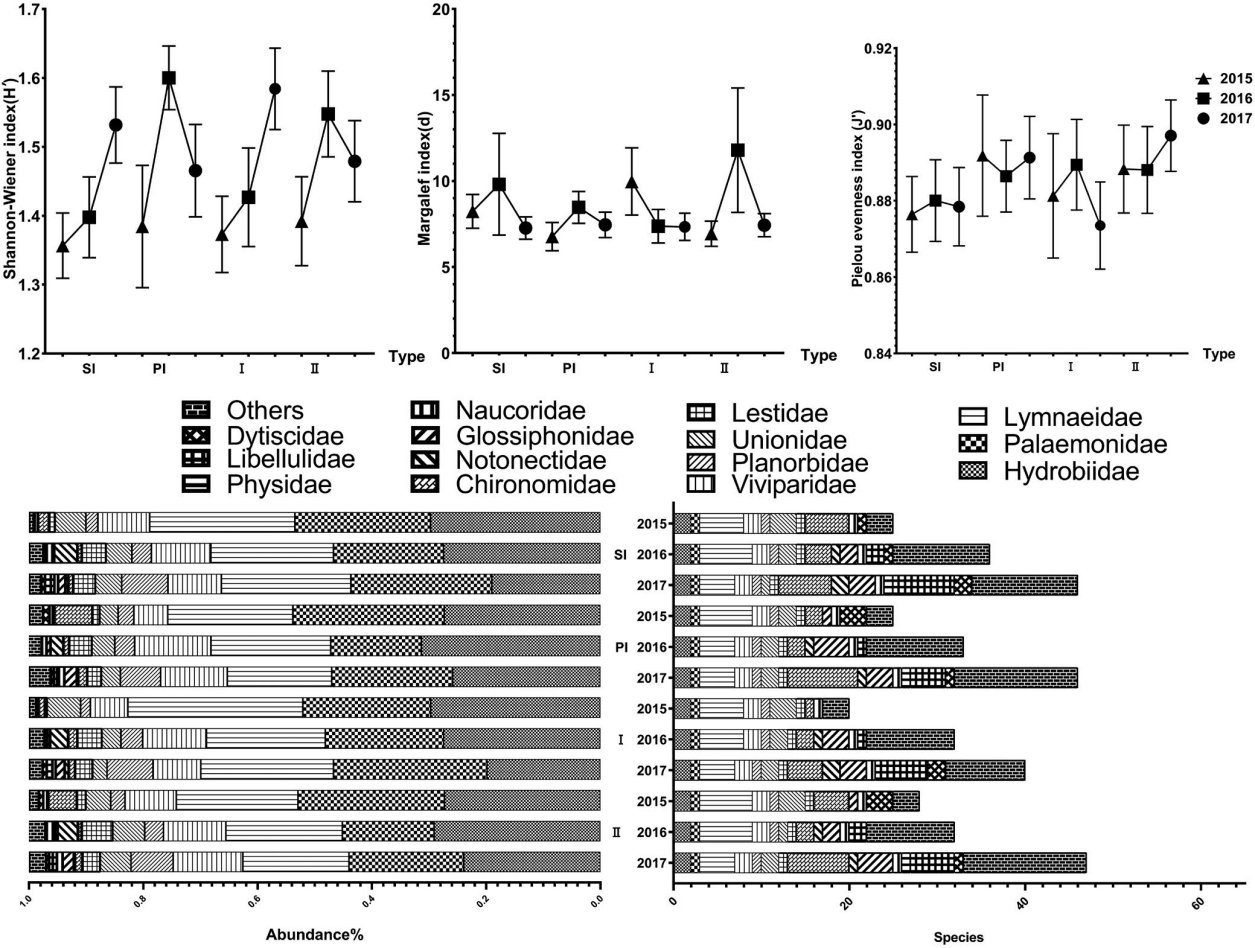
Comparison of the macroinvertebrate composition and biotic indices in the substrate and construction time of the islands (the number of individuals was more than 0.3% of the total) (I: island constructed in 2011; II: island constructed in 2013; PI: pebble substrate island; SI: soil substrate island)

**FIGURE 9:**
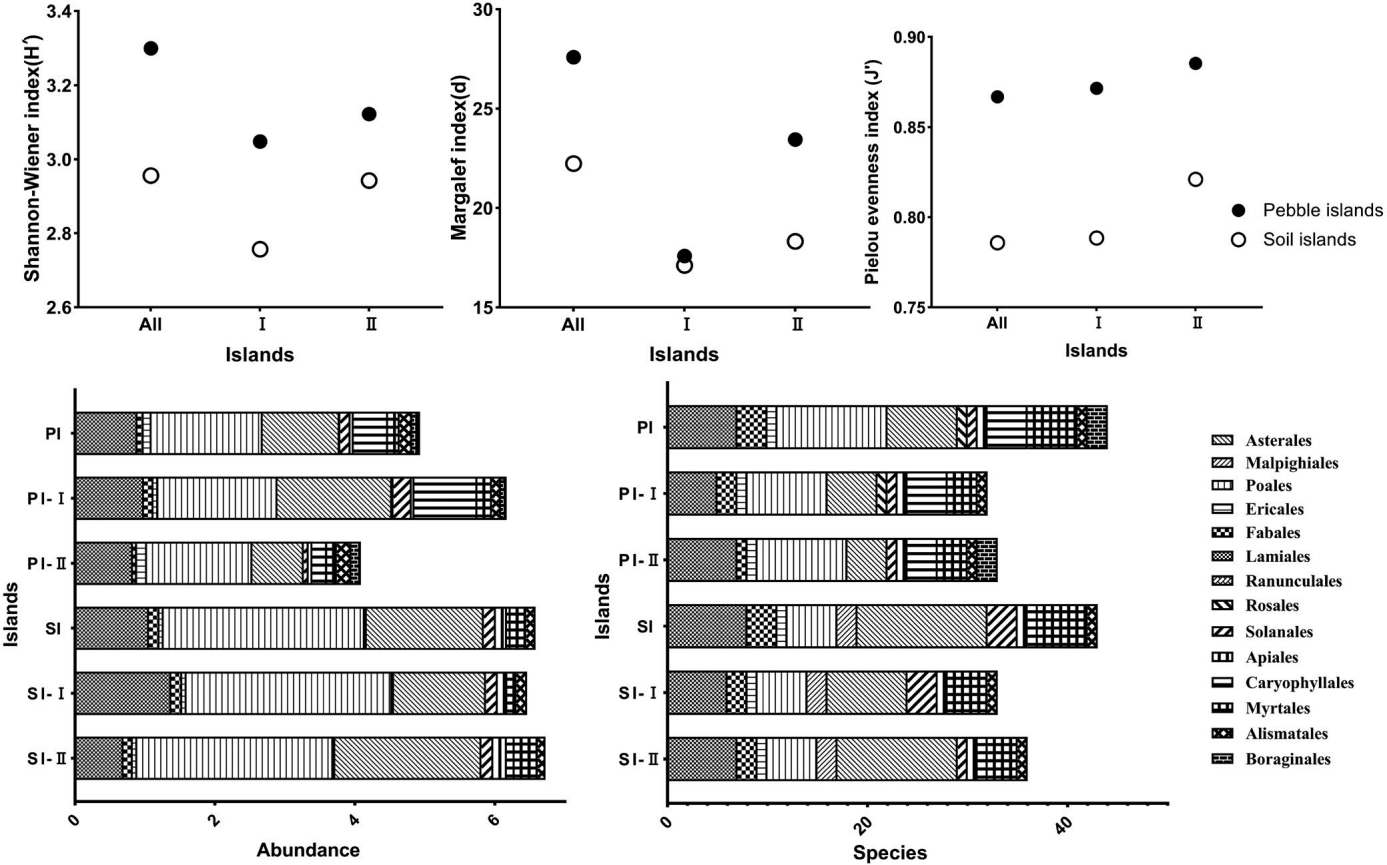
Comparison of the vegetation composition and biotic indices on the islands (I: island constructed in 2011; II: island constructed in 2013; PI: pebble substrate island; SI: soil substrate island)

To explore the change trend in macroinvertebrate diversity with the extension of island construction time, we also compared the community compositions of the islands with two construction ages. The results showed that the composition of macrobenthos communities was different due to the years of island construction (*p* = .001) (Table 3). The abundance of 8 families was significantly different between construction ages (e.g., Unionidae (*p* = .001), Naucoridae (*p* = .007), and Cordulegasteridae (*p* = .046)) (Table 2). 20 of these families are significantly abundant in the younger islands (e.g., Unionidae, Physidae, Naucoridae, Belostomatidae, and Cordulegasteridae). However, 13 families of benthos (e.g., Lymnaeidae, Palaemonidae, Nepidae, Hydrophilidae, and Arachnida) are significantly abundant on the older islands. The species and diversity were higher on the islands that were built relatively later (Figure 8). Compared with the vegetation biodiversity and abundance on these islands, PIs were higher in plant species and biodiversity than SIs although plant abundance was lower. In addition, the vegetation diversity of the later islands is higher than that of the earlier islands (Figure 9).

## 4 DISCUSSION

### 4.1 Effects of artificial ecological islands on macroinvertebrates

The macroinvertebrate community in the park was mainly composed of Mollusca, Palaemonidae, and aquatic insect, possibly because the plant community was mainly composed of *Phragmites australis*, *Typha orientalis*, and *Myriophyllum*. The substrate was mostly silt that was rich in humus, which meet the requirements of some species for dissolved oxygen and organic debris. The park's artificially controlled water flow keeps constant, providing excellent conditions for slow‐moving species, such as Mollusca, to thrive (Chen et al., 2014; Zuo et al., 2016). With the management of farmland around the park, the water quality in the park is relatively clean and hydrophytes grow thickly. The abundances of *Palaemon (Exopalaemon) modestus*, Ephemeroptera, Lestidae, Dytiscidae, and other aquatic insects such as Nepidae, Belostomatidae, and Haliplidae that are suitable for living in aquatic plants also increased. The macroinvertebrate species were abundant and dense, but the biomass was low, which was related to their geographical environment and the short amount of time that had passed since the farmland was converted to wetland. Regarding the increasing trend in the macroinvertebrate diversity, consistent with the works of Du and Lu, the diversity index showed an increasing trend with the extension of time since the construction of conservation engineering projects (Du et al., 2011; Lu et al., 2013). Over time, the ecological effects of these projects become evident. The results of significant difference analysis showed that the construction of artificial islands contributed to the change in the macrobenthos biodiversity. The community structure of macrozoobenthos obviously changed with the change in water depth. Heino (2000) showed a positive correlation between species richness of scraping predators and river depth. Flow velocity is also considered to be an important factor in the habitat of macrobenthos (Leunda et al., 2009). Unfortunately, we do not have data on the wetland park before it was restored. However, in the summer of 2017, Meng (2019) conducted a survey of macrobenthos in Qixinghe Nature Reserve (QXH). QXH is about 40 km from the park (132°05 ‘~132°26’, 46°40 ‘~52’, 200 km^2^). They all belong to the hinterland of Sanjiang Plain and have similar natural environmental factors. Therefore, we can compare QXH and park as the original habitat and the restored habitat in the same wetland. The results showed that there were 25 species (19 families and 10 orders) of macrobenthos in QXH. However, 64 species (30 families and 12 orders) of benthos were found in the park during the same period. Among them, 61 species (30 families, 12 orders) were found on islands and 22 species (12 families, 9 orders) were found in open water. In addition, the deep water of the islands is richer in macrobenthic biodiversity than the open‐water. In conclusion, the ecological island changed the single habitat of benthos in the wetland and increased the gradient change in ecological factors such as shallow water habitat, tidal wetland, and water depth. Although there are some limitations to the role of artificial islands, our results have important implications in the context of biodiversity conservation. This is especially the case where spatial issues need to be considered in the development of wetland ecological restoration strategies (Briers & Biggs, 2007).

First of all, on the one hand, the wetland area of the park becomes undulating after microtopography treatment, and the open‐water area is distributed in a Mosaic shape with the island. Such undulating terrain increases the surface area and soil volume of wetland (Li et al., 2008). It also changes the light and temperature at the bottom of the island, increasing the niche range of plant species, and offering the possibility of increasing benthic biomass and survival rates (Carvalho et al., 2012; Freitas et al., 2011; Wen et al., 2016). On the other hand, from the perspective of functional movement groups, Basommatophora are clingers. They do not like migration, like to adhere to the coastal sand and gravel, so they will inevitably choose rough bottom, slow water habitat. From the perspective of functional feeding groups, the predators in this study are aquatic insects and Glossiphonidae. Predators eat directly from the water, such as plankton or meiofauna (Meng, 2019). The growth of phytoplankton especially needs the promotion of inorganic salts such as nitrogen and phosphorus in the water. Therefore, predators prefer the humus in the mud and sand bottom environment, the presence of vegetation will also provide a part of their food source. And predators are better suited to running water, but fast flow makes it harder for them to inhabit and hide. Primary productivity, phytoplankton, and sedimentary organic matter are affected by water depth changes (Chen et al., 1975, 2014; Du et al., 2011). Therefore, the construction of ecological island is helpful to improve the heterogeneity and diversity of riverbed sediment and water depth, and slow down the flow velocity. This can significantly change the community structure and distribution pattern of macrozoobenthos, thus achieving the purpose of increasing the biodiversity of macrozoobenthos.

Thirdly, wetland substrate (including substrate size, heterogeneity, surface structure, stability, etc.) is the basis of life activities such as the growth and reproduction of macrozoobenthos. It is an important environmental factor affecting the community structure of aquatic invertebrates in wetlands (Duan et al., 2007). The community composition and distribution characteristics of macrozoobenthos are largely influenced by the type and composition of substrate (Beauger et al., 2006; Buss et al., 2004; Heino & Mykrä, 2008). In theory, silt, fine sand, and gravel have poor stability and low heterogeneity, and the biomass and diversity of macrobenthos are lower than those of pebbles, which have complex surface structure and good stability. Therefore, when constructing PI, the park's designers chose larger stones as the covering, rather than fine sand or small stones to ensure the stability of the matrix. In this study, SI was surrounded with many hydrophytes, such as *Phragmites australis*, *Typha*, and floating grass, with loose debris at the bottom and abundant organic matter. These conditions provide a place for macrobenthos to feed, breed, and avoid predators, and a stable substrate reduces the impact of water‐level changes on macrobenthos (Duan et al., 2007). Therefore, compared with PIs, with less vegetation and humus, SIs are more suitable for survival.

Finally, the biomass of dominant species such as *Typha* and *Phragmites australis* increased year by year, leading to the singleness of hydrophyte on the island. *Phragmites australis* are shallow‐rooted scattered plants that have a strong ability to secrete oxygen from their roots, and these conditions can meet the respiratory needs of Mollusca, such as Gastropoda, which require high dissolved oxygen levels. However, these plants have high growth density, which makes the stems and leaves difficult to decompose (Zuo et al., 2016), and produce less organic detritus. As a result, the abundance and diversity of macroinvertebrate species on the older islands are lower than on the younger islands. Plants are more likely to survive on soil islands (high abundance), but as soil islands age, plants tend to become more homogeneous (Molles, 2016). However, the stone island has been scoured by water for a long time, which is not conducive to the intensive growth of plants, making plants more dispersed and more diverse. For example, SIMPER analysis showed that the three most contributing species on the island, which was constructed in 2011, were *Phragmites australis* (35.19%), *Scutellaria scordifolia* (16.94%), and *Inula japonica* (15.80%). However, the top three plants that contributed most to the construction of islands in 2013 were *Phragmites australis* (20.29%), *Carex bohemica* (20.27%), and *Calamagrostis epigeios* (10.93%). Due to mowing a year ago, soil island J has the highest plant biodiversity, species, and abundance compared with other soil islands. The number of macroinvertebrate species was negatively correlated with plant abundance (*r* = −.689, *p* = .04), and aging of islands leads to a loss of attractiveness for plant and birds (Scarton et al., 2013). Therefore, it is recommended that wetland parks conduct reed‐cutting work regularly to promote the increasing diversification of hydrophytes.

### 4.2 Effects of artificial ecological islands on other species

The change in topography can affect the formation of landscape pattern in the park. Water and soil conditions also change as the landscape changes. Species suitable for particular habitats will colonize the park, changing the plant and animal species and distribution in the park. Among the vertebrates in the park, there are many species of fish and waterbirds, and few amphibians and reptiles.

The distribution of fish is mainly caused by the heterogeneity of environmental factors, including water depth, temperature, salinity, dissolved oxygen, turbidity, water system, and substrate type (Amara & Paul, 2003; Andres et al., 2004; Wantiez et al., 1996). In addition, increased biodiversity of hydrophytes and macrobenthos as food sources directly affects fish diversity (Hornung & Foote, 2006). Therefore, through the construction of different water depth environments and terrains, the habitat factors of fish have gradient change and heterogeneity, thus increasing the biodiversity of fish.

Water area, bare land, and vegetation are three important habitat factors that affect the biodiversity of waterbirds in natural wetlands (Davis, 1998; Mark & Sarah, 1994). Studies have shown that the number of Charadriidae is highest when the vegetation coverage is between 10% and 20% (Tang & Lu, 2002). The interaction among hydrophyte, macrobenthos, and waterbirds in freshwater wetlands is a complex interdependency (Patra et al., 2010). The shallow water areas of the islands may have increased the temperature of the water and the light penetrating the water column, thereby promoting the growth of aquatic plants and increasing the food source for birds (van den Berg et al., 1997; Zimmer et al., 2000). Aquatic vegetation provides a carbohydrate‐rich food source for waterbirds, which is important for autumn aggregation and migration (Baldassarre & Bolen, 2006; Baschuk, 2010). Simultaneously, vegetation can also provide support and concealment for the movement of birds (Baschuk, 2010; Desrochers & Ankney, 1986; Lor, 2007; Rehm & Baldassarre, 2007; Rehm & Baldassarre, 2007). In addition, high density hydrophyte increases the amount of habitat available for invertebrates, which may increase the abundance of macrobenthos. Macrobenthos affect the dynamics of bird communities by decomposing vegetation and changing its habitat (Backwell et al., 1998; Murkin & Kadlec, 1986; Patra et al., 2010; Voigts, 1973; Wilson, 1990). Pearson correlation analysis in this study showed that the diversity of waterbirds and macrobenthos (H’) was negatively correlated (*r* = −.997, *p* = .05). The number of tourists was negatively correlated with the diversity (*r*_H'_ = −1.000) and richness (*r*_d_ = −.999) of waterbirds. This may be because the number of visitors increases with the popularity of the park. The noise of human activities interferes with the foraging behavior of birds, reduces the space of their activities, and has a driving effect on them.

Habitat selection by waterbirds is strongly related to the water depth and food availability (Boshoff et al., 1991; Velasquez, 1992). Water depth limits the feeding behavior, and energy expenditure of waterbirds affects the availability of food and determines habitat utilization (Ma et al., 2010; Murkin et al., 1997). For example, the length of the waders' beaks and legs limits the foraging range in the shallows (Nolet et al., 2002). The optimal water level for Anasanas and Charadriws is between 10–20 cm and 15–20 cm, respectively (Elphick & Oring, 1998; Taft et al., 2002). In natural mudflats, the distribution of waders and recreational birds among various habitat elements depends on the abundance of their bait (Wilson, 1990). For example, the breeding habitat of *Egretta Garzetta* requires open shallow water (10–30 cm) with abundant food and high vegetation coverage (40–60%) (Thompson, 1979). Anatidae, Podicipedidae, and Rallidae generally feed on seeds, fish, and macrobenthos, mostly use deepwater areas. The construction of artificial islands creates more vegetation/water edges, which can increase the number of available foraging sites and reduce competition between species in the same ecological niche. This ensures niche differentiation and stable coexistence of waterfowl in the community (Colwell & Taft, 2000; Shao et al., 2016). Similar results were obtained from experiments conducted by Gao and Lu (2008) on wetland bird habitat construction. Habitat units and biological component diversity (vegetation, macrobenthos, fish, birds, etc.) of the ecosystem in experimental areas with artificial islands were significantly increased compared with those in areas without islands. Therefore, the ecological engineering ideas and techniques for the construction of suitable habitat for animals in this study are feasible. The construction of artificial islands increases the microhabitat heterogeneity and water‐level gradient. By increasing the biodiversity of macrobenthos to attract more predators, such as fish and waterfowl, and ultimately to increase the biodiversity of the ecosystem, it is of great value in the restoration and reconstruction of wetland ecology. Notably, waterbird avoided islands with high *Typha* density in this study. It is possible that too much vegetation would hinder the movement of waterbird on islands (Baschuk, 2010). Consider this, it is recommended that the park should regularly control and manage the vegetation on the island (Xiong et al., 2007).

The predominant mammals in the park are Rodentia (e.g., *Cricetulus barabensis*). The diversity of rodents' diet is related to plants. Simultaneously, the microclimate of vegetation formation is an important factor affecting their habitat (Zhou et al., 1982). In the park, Rodentia mainly live on the high slopes of grasslands where the water level is low, and feed on the green parts or seeds of herbaceous plants. The construction of the ecological island will attract Rodentia to settle and multiply. The dominant species of Amphibia are *Bufo raddei* and *Rana amurensis*. Adults live on land, but egg hatching and tadpole development must be completed in water. The island's intersecting zone facilitates the seasonal migration of Amphibia between water and land (Li, Gu, et al., 2008). Most Reptilia like to drink, forage, and breed at the edge of the water, and the water‐land junction area around the island is an ideal habitat environment for Reptilia.

Given the urge to conserve biodiversity, especially in the context of climate change, ecological managers should not only protect existing species, but also consider improving the biodiversity of ecosystems by constructing ecological islands to increase the diversity of habitats. Therefore, the management of wetlands (distribution of wildlife and plant communities) should integrate the potential of these biodiversities, especially in human‐dominated landscapes. During the construction of water level management, the park provides a mosaic of deep and shallow wetlands, staggers the water level of the wetland complex. Artificial islands could be used as shallow wetland habitats, while the open‐water area could be used as deepwater habitats, thus creating a diverse wetland habitat. The construction of artificial ecological islands contributes to the improvement of the entire wetland ecosystem in the park to coordinate the movement of energy, matter, and organisms in the landscape. It promotes the material circulation, energy flow, and information transmission of the wetland ecosystem, improves the stability of the system, and enhances the ability to resist external interference. Our results contribute to a better understanding of the positive role of artificial islands in increasing biodiversity in the wetland ecosystem. It is not difficult to predict that with the passage of time, the ecological benefits of artificial ecological islands will become more prominent, which can provide reference for the wetland restoration work in the world.

## CONFLICT OF INTEREST

None declared.

## AUTHOR CONTRIBUTIONS

**Zi‐Ao Yuan:** Data curation (lead); Formal analysis (lead); Investigation (lead); Writing‐original draft (lead). **Xin‐xin Liu:** Investigation (supporting). **Hai‐rong Du:** Investigation (supporting). **Ming‐Hai Zhang:** Conceptualization (lead); Funding acquisition (lead); Project administration (lead); Writing‐review & editing (lead).

## Data Availability

All authors of this submission have read and understand the journal's policy on completing the Open access Agreement. My raw data were uploaded to the Dryad database with the link: https://doi.org/10.5061/dryad.x95x69pdg.
